# Verbal memory and hippocampal volume predict subsequent fornix microstructure in those at risk for Alzheimer’s disease

**DOI:** 10.1007/s11682-019-00183-8

**Published:** 2019-09-07

**Authors:** Junhong Yu, Tatia M. C. Lee, for the Alzheimer’s Disease Neuroimaging Initiative

**Affiliations:** 1 The State Key Laboratory of Brain and Cognitive Sciences, The University of Hong Kong, Pokfulam, Hong Kong; 2 Laboratory of Neuropsychology, The University of Hong Kong, Rm 656, Jockey Club Tower, Pokfulam Road, Hong Kong, Hong Kong; 3 Institute of Clinical Neuropsychology, The University of Hong Kong, Pokfulam, Hong Kong; 4 Guangdong-Hong Kong-Macao Greater Bay Area Center for Brain Science and Brain-Inspired Intelligence, Guangzhou, China

**Keywords:** Alzheimer’s disease, ADNI, Fornix, Hippocampus, Memory, Longitudinal

## Abstract

While strong cross-sectional evidence supported the use of fornix microstructure as a marker for detecting Alzheimer’s disease (AD), longitudinal data remains inconclusive on the sequential nature of fornix microstructure abnormalities and AD progression. An unequivocal longitudinal relationship between fornix microstructure and markers of AD progression –memory impairment and hippocampal atrophy, must be established to validate fornix microstructure as a marker of AD progression. We included 115 participants from the Alzheimer’s Disease Neuroimaging Initiative across the non-demented AD spectrum— defined as those who had at least one AD risk marker at baseline (e.g., mild cognitive impairment (MCI) due to AD diagnosis, amyloid or ApoE4 positivity) and/or ‘cognitively normal individuals who converted to MCI due to AD or AD, with structural and diffusion tensor imaging scans at baseline and two years follow-up. Hippocampal volumes (HV), fractional anisotropy (FA) and mean diffusivity (MD) in the fornix were extracted. Memory was indexed via composite scores of verbal memory tests. Structural equation models tested the bidirectional cross-lagged effects of fornix microstructure, memory, and HV. Impaired memory and smaller HV at baseline significantly predicted worse fornix microstructure (decreased FA and increased MD) two years later. Baseline fornix microstructure was not associated with subsequent changes in memory and HV. Fornix microstructure is compromised likely at a later stage, where significant decline in memory and hippocampal atrophy have occurred. This limits the utility of fornix microstructure in the early detection of AD. Our findings inform the possible pathophysiology and refined the use of AD neural markers.

**Electronic supplementary material:**

The online version of this article (10.1007/s11682-019-00183-8) contains supplementary material, which is available to authorized users.

## Introduction

Alzheimer’s disease (AD) is a chronic neurodegenerative condition characterized by progressive memory impairment. Early detection and intervention are crucial to maximize prognostic outcomes. To this end, non-invasive neural markers have been suggested to facilitate early detection (Sperling et al. 2011). One candidate marker that has garnered recent interest is the white matter (WM) microstructure of the fornix, which can be observed via the diffusion tensor imaging (DTI) indices of fractional anisotropy (FA) and mean diffusivity (MD). The fornix is a bundle of WM fibers that connects the hippocampus to subcortical structures. Due to this structural association with the hippocampus — a region that is crucially implicated in both short-term and long-term memory, it has been widely studied in AD. Cross-sectional studies using DTI have revealed significantly altered WM microstructure in the fornix as indicated by decreased FA and increased MD, among individuals with AD (Oishi and Lyketsos 2014), as well as its prodromal amnestic mild cognitive impairment (aMCI) stage (Yu et al. 2017). Whilst these findings provide strong preliminary evidence, their cross-sectional nature limits their ability to illustrate the sequential nature of the relationship between progressive memory decline and changes in fornix microstructure, which would be an important consideration in using fornix DTI measures as a marker of AD progression.

The limited longitudinal findings on fornix microstructure and memory impairment are however, less consistent and have failed to arrive at a satisfactory conclusion regarding the precise nature of the relationship between fornix microstructure and memory impairment. A few studies have looked at how baseline fornix WM indices predict subsequent memory outcomes. Mielke et al. (2012) found fornix FA, but not MD, predicted subsequent memory decline across a 2.5-year period among participants with aMCI. In a study of healthy participants who converted to aMCI two years later (Zhuang et al. 2012), these converters had significantly reduced fornix FA at baseline. Fletcher et al. (2013) on the other hand, found neither baseline fornix FA or MD among healthy participants predicted their subsequent conversion to mild cognitive impairment (MCI; unclear if they were of the amnestic subtype) or AD. Nevertheless, they found baseline fornix axial diffusivity, but not radial diffusivity, to be related to subsequent conversion to MCI or AD. Two other studies examined the reverse association (i.e., how memory impairment at baseline predicted subsequent changes in fornix WM indices). Teipel et al. (2010) compared changes in fornix FA across a 13-to-16 months period among participants identified as aMCI and healthy controls at baseline. They noted that there was a general decrease in fornix FA across the entire studied sample. However, there was not a significant time by group interaction effect to suggest that the decline in fornix FA is specific to aMCI. In another study, fornix MD increased significantly over a 1-year period among participants with aMCI, relative to healthy controls. Interestingly, such differences between participants with AD and healthy controls were not significant despite significant baseline differences. Furthermore, none of the between-group differences in longitudinal FA changes were statistically significant (Nowrangi et al. 2013).

These longitudinal studies were subject to some methodological limitations. Firstly, the majority of these studies (Fletcher et al. 2013; Nowrangi et al. 2013; Teipel et al. 2010; Zhuang et al. 2012) analyzed binary memory-related diagnostic outcomes, instead of memory test scores on a continuum. While they intended to study these diagnoses rather than memory per se, this binary approach may not adequately capture the variability in memory impairment across the AD spectrum, and thus may risk oversimplifying the relationship between memory impairment and fornix WM changes. Understandably, the small sample sizes of these studies may not make it practical to operationalize memory impairment on a continuum of test scores. Secondly, studies (Fletcher et al. 2013; Mielke et al. 2012; Zhuang et al. 2012) that examined how baseline fornix WM indices may predict subsequent memory-related outcomes did not control for baseline levels of memory impairment. It is possible that those participants with worse fornix microstructure relative to others at baseline may also start off with worse memory impairment. As a result, they will naturally present with worse memory impairment at follow-up, simply because of their lower baseline levels.

If such disruptions in fornix microstructure do indeed occur in the context of AD-related memory impairment, there may be two possible explanations. According to the conventional amyloid cascade hypothesis, memory impairment in AD has been theorized to be a result of amyloid-beta mediated neuronal death (Hardy and Selkoe 2002). Kantarci (2014) suggested that the death of hippocampal neurons would subsequently lead to the degeneration of the fornix axons, especially since most of the axonal projections carried by the fornix originate from the hippocampus. Alternatively, memory loss may not always be a result of neuronal loss (Caso et al. 2016); a more recent theory implicates immune-system mediated neuroinflammatory processes in the pathogenesis of AD (Heppner et al. 2015). Neuroinflammation observable via WM microstructural changes may also contribute to memory impairment (Caso et al. 2016). There is little experimental or longitudinal evidence to support either explanation. Unraveling the longitudinal relationship between hippocampal atrophy and fornix microstructure would be a useful first step in evaluating these theories.

In light of the above gaps in knowledge, the current study examined the longitudinal relationship between fornix microstructure and other non-invasive markers of AD progression such as verbal memory and hippocampal volume. The aim was to examine the antecedents and consequences of such fornix microstructural changes in the onset of AD. This information will be highly relevant in evaluating the validity of using fornix microstructure in the early detection of AD. Additionally, these findings are important to understand the pathophysiological mechanisms involved in the prodromal stages of the disease. Using the longitudinal data from the Alzheimer’s Disease Neuroimaging Initiative (ADNI), we examined auto-regressive cross-lagged models of fornix microstructure and memory impairment, as well as that of fornix microstructure and hippocampal atrophy. In the former, in line with the reviewed evidence, we hypothesized a bidirectional relationship. Specifically, worse fornix microstructure, observed via decreased FA and increased MD, would be associated with subsequent verbal memory impairments and greater verbal memory impairments would predict worse fornix microstructure subsequently. As for the latter model, we hypothesized that smaller hippocampal volumes at baseline would predict worse fornix microstructure at follow-up, in line with Kantarci's (2014) explanations.

## Materials and methods

Data used in the preparation of this article were obtained from the ADNI database (adni.loni.usc.edu). The ADNI was launched in 2003 as a public-private partnership, led by Principal Investigator Michael W. Weiner, MD. The primary goal of ADNI has been to test whether serial magnetic resonance imaging (MRI), positron emission tomography (PET), other biological markers, and clinical and neuropsychological assessments can be combined to measure the progression of MCI and early AD. Ethical approval for the ADNI study was obtained by the ADNI investigators. For up-to-date information, see www.adni-info.org.

### Participants

Participants on the ADNI database with valid structural T1 and/or DTI scans at the baseline and two-year follow-up, specifically from the ADNI GO and ADNI 2 phases, were potential subjects of this study. Informed consent was obtained from all individual participants included in the study. Given that cross-lagged analyses depends not just on within subject variance, but that of between subject as well, it is important to capture as much variance as posssible across the heterogenous spectrum AD spectrum. Nevertheless, if the sample is too heterogenous, the interpretation and generalization of the results would be difficult. Hence there is a need to balance between maximizing variance across the included sample and also interpretability of the results, as such we limited the heterogeneity of the participants by excluding participants at both ends of the AD spectrum— cognitive normal participants without any AD related biomarkers and those who were already diagnosed with AD. Furthermore, the exclusions of the former group is further justified to enable the findings of the current study to be as specific to AD as possible and the exclusion of the latter group is further justified by the fact that we are primarily interested in studying the early phases of AD and not individuals who are already afflicted with AD. To these ends we restricted the participants’ inclusion to those of the non-demented AD spectrum as defined by one or more of the following criteria:

- Being diagnosed with MCI due to AD at baseline
- Cognitively normal participants at baseline who converted to probable AD or MCI due to AD at subsequent time points (including those beyond the follow-up if such data is available in the ADNI dataset)
- Cerebrospinal fluid (CSF) amyloid-beta positive (Aβ+), as defined by having CSF amyloid-beta 1 to 42 peptide (Aβ_1–42_) concentration levels ≤192 pg/mL
- Having at least a single copy of the Apolipoprotein ε4 gene (APOε4+).

The diagnosis of MCI due to AD was made by a physician during the participant’s in-clinic visit. Briefly, the MCI diagnosis has to fulfill all of the following criteria: presence of a subjective memory concern, scores of Logical Memory II from the Wechsler Memory Scale below education-adjusted cutoffs, Mini-mental Status Examination (MMSE) scores above 23 and Clinical Dementia Rating (CDR) of 0.5. Subsequently, the suspected cause of the MCI was determined to be due to AD after ruling out all other possible etiologies. Using the same tests, the diagnosis of probable AD was made according to the National Institute of Neurological and Communication Disorders/Alzheimer’s Disease and Related Disorders Association criteria. This criteria for AD was similar to MCI due to AD in terms of requiring a subjective memory concern, Logical Memory II scores below similar cutoffs and the ruling out of all other possible etiologies. On top of that, the participant is required to have an MMSE score between (inclusive) 20 to 26 and a CDR of 0.5 or 1.0. Detailed cut-off scores of the different tests and other criteria for these diagnoses were reported in the ADNI procedures manual (https://adni.loni.usc.edu/wp-content/uploads/2008/07/adni2-procedures-manual.pdf). The collection, processing, and storage of CSF were carried out according to the ADNI procedures manual; CSF samples were acquired at the ADNI clinical centers, frozen at −80°c and shipped for analysis to the ADNI Biomarker Core. CSF Aβ_1–42_ concentration values were obtained from analyzing these samples using the xMAP Luminex platform and Fujirebio AlzBio3 immunoassay kit at the UPenn/ADNI Biomarker Laboratory, according to the kit’s manufacturer instructions. The cutoff of CSF Aβ_1–42_ ≤ 192 pg/mL had high sensitivity (96%) and specificity (77%) in classifying AD and cognitively normal cases in an ADNI sample (Shaw et al. 2009).

**Fig. 1:**
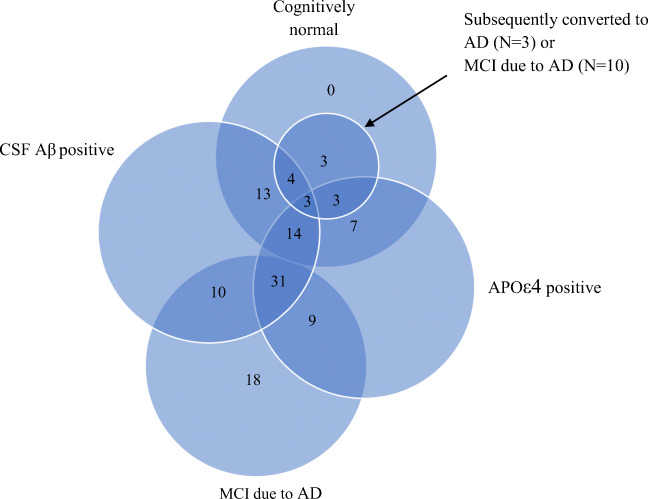
Venn diagram showing the included participants with the various AD-spectrum related characteristics. CSF Aβ = Cerebrospinal fluid amyloid-beta; AD = Alzheimer’s disease; MCI = Mild cognitive impairment

Next, we further removed three participants due to image quality issues. The remaining 115 participants (54 females) were included in this study. The number of participant with the various AD-spectrum characteristics are presented in a venn diagram in Fig. 1. Among the 115 participants, 98 and 112 participants had valid DTI and T1 structural scans, respectively, at both time points. The characteristics of these participants are presented in Table 1.

**Table 1:** Characteristics of included participants

	Mean (SD)/Frequency (%)
Age_baseline_	73.1 (6.9)
Gender	
Male	61 (53.0%)
Female	54 (47.0%)
Years of education	16.0 (2.6)
Neurocognitive diagnosis	
Cognitively normal at baseline	47 (40.9%)
Remained at cognitively normal at follow-up^a^	34 (72.3%)
Converted to MCI due to AD at follow-up	10 (21.3%)
Converted to AD at follow-up	3 (6.4%)
MCI due to AD at baseline	68 (59.1%)
Remained at MCI due to AD at follow-up	52 (76.5%)
Converted to AD at follow-up	16 (23.5%)
MMSE scores_baseline_	28.0 (1.8)
Ethnicity	
Caucasian	107 (93.0%)
Native American	1 (.9%)
Asian American	3 (2.6%)
African American	4 (3.5%)

SD, standard deviation. MCI due to AD = Mild cognitive impairment due to Alzheimer’s disease

^a^Among these stable cognitively normal participants, 27 are CSF Aβ positive and 21 are APOε4 positive

### Memory scores

We used the ADNI-MEM score (Crane et al. 2012) – a composite measure of multiple verbal memory tests to index memory ability among the participants. Briefly, item scores from the Rey Auditory Verbal Learning Test, AD Assessment Scale–Cognitive Subscale, word recall (3 words) of the MMSE and the Wechsler Logical Memory Scale II were loaded on to this ADNI-MEM composite score.

### MRI acquisition

Participants were scanned with General Electric Healthcare 3 T scanners at various participating sites. T1-weighted images were acquired using a 3D IR-SPGR protocol (TE = 3.036 s; TR = 7.34 s; TI = 400 ms; 256 × 256 matrix; 196 sagittal slices; in-plane resolution = 1.0156 mm; slice thickness = 1.2 mm). DTI images were acquired with an EPI SE protocol (TE = 90 ms; TR = 9.05 s; 256 × 256 matrix, 59 axial slices, in-plane resolution = 1.37 mm; slice thickness = 2.7 mm; diffusion-weighted volumes in 41 directions; b-value = 1000 s/mm^2^). Further information regarding these protocols is available online (http://adni.loni.usc.edu/methods/documents/mri-protocols/).

### Image preprocessing

The raw diffusion images were skull-stripped and corrected for eddy current and subject movement using FMRIB Software Library (FSL 5.0.8). Diffusion tensors were then generated for each participant. We used an unbiased approach (Keihaninejad et al. 2013) for the longitudinal registration of the diffusion tensors. For each subject, a within-subject template was generated by computing the initial average template as a Log-Euclidean mean of the baseline and follow-up diffusion tensors. This template was iteratively refined; the diffusion tensors were registered to the template, and a refined average template was similarly computed from the two registered diffusion tensors for the next iteration. The process was repeated until the difference between templates from consecutive iterations became sufficiently small; first with affine and then with non-linear registrations. Next, a study template was created from all the final average templates in the within-subject space using the same iterative method. Then, FA and MD maps were generated from each registered tensor image in the study template space. These preprocessing steps were carried out using the diffusion tensor imaging toolkit (DTI-TK). Subsequently, a mean FA skeleton representing the centers of fiber tracts common to all participants, thresholded at FA > .30, was created and then projected onto the FA and MD maps prior to the region-of-interests (ROI) extraction.

The structural T1 images were preprocessed longitudinally using the Computational Anatomy Toolbox (CAT12), as implemented in SPM12. Baseline and follow-up T1 images within a subject were rigidly realigned to correct for differences in head position and a subject-specific average template was computed and used as reference in a subsequent realignment of the baseline and follow-up T1 images. The realigned images were segmented into gray matter (GM), WM and CSF, as well as corrected for signal inhomogeneities with reference to the subject average template. These GM and WM images were used to estimate spatial normalization deformation fields using the high dimensional Diffeomorphic Anatomic Registration Through Exponentiated Lie Algebra (DARTEL) warping algorithm (Ashburner 2007) which were subsequently applied to the individual bias-corrected tissue images to produce affine registered GM and WM images. A study-specific DARTEL template was created using these affine registered images from all participants. This longitudinal registration pipeline was then repeated using this study specific DARTEL template to obtain the final modulated images.

The preprocessed structural T1 and diffusion tensor imaging (DTI) images were then visually inspected for quality. Among the three excluded for image quality issues, two had excessive ghosting effects. As for the third, the DTI acquisition appeared to be incomplete or interrupted; there were missing diffusion directions which resulted in an extremely distorted DTI image. Finally, we used the recently developed HarP hippocampal (Wolf et al. 2017) and younger-older fornix templates (Brown et al. 2017) to extract the hippocampal volumes (HV) as well as fornix FA and MD values. Both ROI templates have been validated in the ADNI sample (Brown et al. 2017; Wolf et al. 2017). The ROI templates were registered to their respective mean study templates prior to the ROI extraction. For each participant, the ROI alignment was visually inspected by overlaying the hippocampus and fornix ROI templates on to their registered T1 and FA images, respectively. Fig. S1 and S2 illustrate the overlays of these ROIs on the mean study images, and the amount of non-zero voxels overlap across volumes. The extracted HVs were then adjusted for total intracranial volume (TIV) using the formula: adjusted HV = HV - b(TIV - TIV_mean_), where b is the regression coefficient for HV against TIV and TIV_mean_ is the average TIV of the sample (Buckner et al. 2004).

### Statistical analyses

Auto-regressive cross-lagged models (Anderson and Kida 1982) were implemented to examine the longitudinal associations between fornix WM indices and ADNI-MEM, as well as, between fornix WM indices and HV. In these models, age, sex and education were included as covariates. These models were analyzed via structural equation modeling (SEM) using a robust maximum likelihood estimator. Missing data were assumed to be missing at random; as such, the data was handled with full information maximum likelihood to maximize all available data. These analyses were carried out with the R package lavaan (Rosseel 2012), in R 3.4.0.

The Comparative Fit Index (CFI) and Standardized Root Mean square Residual (SRMR) were used to assess model fit. The root mean square error of approximation (RMSEA) and chi-square statistics were not used because the relatively small sample size (for an SEM) and degrees of freedom (df = 1) in the current study can significantly bias these estimates (Jackson 2003; Kenny et al. 2015). CFI and SRMR values ≥ .95 and ≤ .10 (Hu and Bentler 1999), respectively, are indicative of acceptable fit. Differences across time in the studied variables were assessed using paired-samples T-tests. Correlations between variables were examined using Pearson correlation coefficients. Statistical significance was set at *p* < .05.

## Results

### Descriptive statistics, bivariate correlations and model fit

Table 2 presents the correlation matrix of all studied continuous variables. Within each of the cross-sectional time points, fornix FA and MD were significantly associated with HV and ADNI-MEM. Table 3 presents the descriptive statistics of the longitudinal measures. There were significant changes in the HV (*t* = 7.78; *p* < .001) as well as fornix FA (*t* = 5.89; *p* < .001) and MD (*t* = 4.27; *p* < .001) in their expected directions, the decline in ADNI-MEM scores was marginally significant (*t* = 1.81; *p* < .073) the violin plots of these measures are shown in fig. S3 in the supplementary materials. The fit indices of all examined SEM models are presented in Table 4. In general, all models had, at least, acceptable levels of fit.

**Table 2:** Correlation matrix of all studied continuous variables

Variable	Age baseline	Edu.	Mem. baseline	Mem. follow-up	FA baseline	FA follow-up	MD baseline	MD follow-up	HV baseline
Age_baseline_									
Edu.	−.08								
Mem._baseline_	−.11	.10							
Mem._follow-up_	−.06	.05	.86***						
FA_baseline_	−.48***	.01	.23*	.18					
FA_follow-up_	−.56***	−.01	.31**	.26**	.87***				
MD_baseline_	.53***	.15	−.23*	−.19	−.89***	−.85***			
MD_follow-up_	.56***	.10	−.28**	−.24*	−.82***	−.93***	.93***		
HV_baseline_	−.49***	<.01	.38***	.40***	.59***	.68***	−.59***	−.65***	
HV_follow-up_	−.49***	.03	.43***	.43***	.60***	.71***	−.60***	−.67***	.93***

Edu, Education in years; Mem, memory composite scores (ADNI-MEM); FA, Fractional Anisotropy of fornix; MD, Mean Diffusivity of fornix; HV, Hippocampal volume (adjusted for intracranial volume). *p < .05; **p < .01; ***p < .001

**Table 3:** Descriptive statistics of longitudinal measures

	ADNI-MEM		Fornix			T1 Structural		
	Valid N	M (SD)	Valid N	FA M (SD)	MD (10^−3^ mm^2^/s) M (SD)	Valid N	HV (cm^3^) M (SD)	TIV (cm^3^) M (SD)
Baseline	115	.417 (.693)	98	.350 (.048)	1.399 (.202)	112	7.243 (1.031)	1477 (131)
Follow-up	115	.339 (.897)	98	.334 (.053)	1.433 (.216)	112	6.926 (1.177)	1479 (135)
Follow-up - baseline	115	−.078 (.465)	98	−.016(.026)	.034 (.079)	112	−.316 (.145)	2.521 (26.3)

ADNI-MEM, memory composite scores; FA, Fractional Anisotropy; MD, Mean Diffusivity; HV, Hippocampal volume (adjusted for intracranial volume); TIV, intracranial volume; M, Mean; SD, Standard Deviation

**Table 4:** Fit indices of all examined SEM models

Model	CFI	SRMR
Fornix FA ↔ ADNI-MEM	.993	.039
Fornix MD ↔ ADNI-MEM	.994	.039
Fornix FA ↔ HV	.950	.095
Fornix MD ↔ HV	.960	.093

CFI, Comparative Fit Index; SRMR, Standardized Root Mean Square residual; FA, Fractional Anisotropy; MD, Mean Diffusivity; HV, Hippocampal volume (adjusted for intracranial volume); ADNI-MEM, memory composite scores

### Cross-lagged analyses of fornix WM and memory

**Fig. 2:**
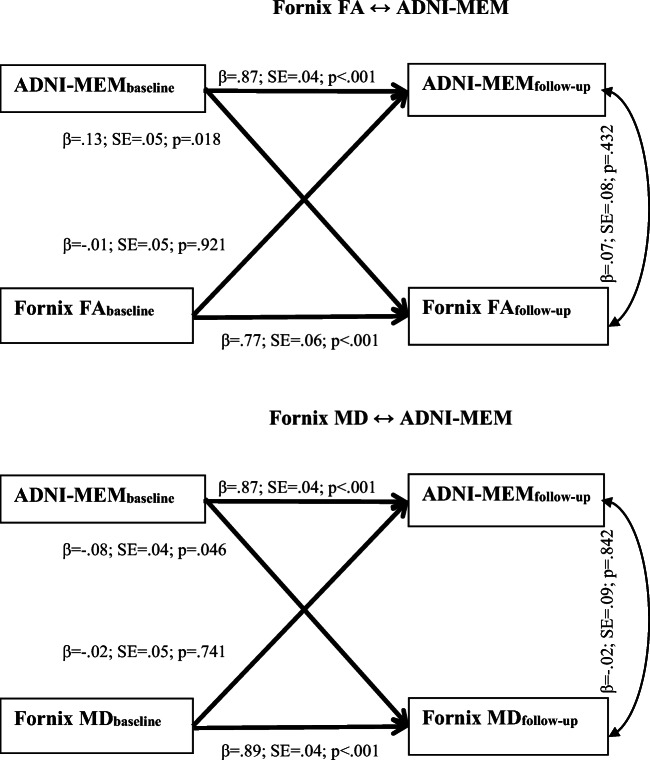
Robust Maximum likelihood estimation of the cross-lagged effects between extracted regions of interest and ADNI-MEM, with age, sex and education included as covariates. Straight lines represent regression paths. Curve line represents residual covariance. FA = Fractional Anisotropy; MD = Mean Diffusivity; β = standardized coefficients; SE = Standard Error

The results of the cross-lagged analyses between fornix WM indices and ADNI-MEM are presented in Fig. 2. The autoregressive paths in both models were significant; that is, ADNI-MEM scores at baseline significantly predicted its scores at follow-up, and the fornix WM indices at baseline significantly predicted their respective follow-up measures (*β*s > .77; *p*s < .001). Next, ADNI-MEM at baseline significantly predicted fornix FA and MD at follow-up (*p*s ≤ .046) in their expected directions, after controlling for baseline fornix FA and MD respectively. However, the reverse associations were not significant; Fornix FA and MD at baseline did not significantly predict ADNI-MEM at follow-up (*p*s ≥ .741), after controlling for baseline ADNI-MEM. For the purpose of comparison, a similar cross-lagged model was carried out on HV and ADNI-MEM; smaller baseline HVs were significantly associated with lower ADNI-MEM scores at follow-up, after controlling for baseline ADNI-MEM scores (*β* = .14; SE = .05; *p* = .007; see fig. S4 in the supplemental materials).

### Cross-lagged analyses of HV and fornix measures

**Fig. 3:**
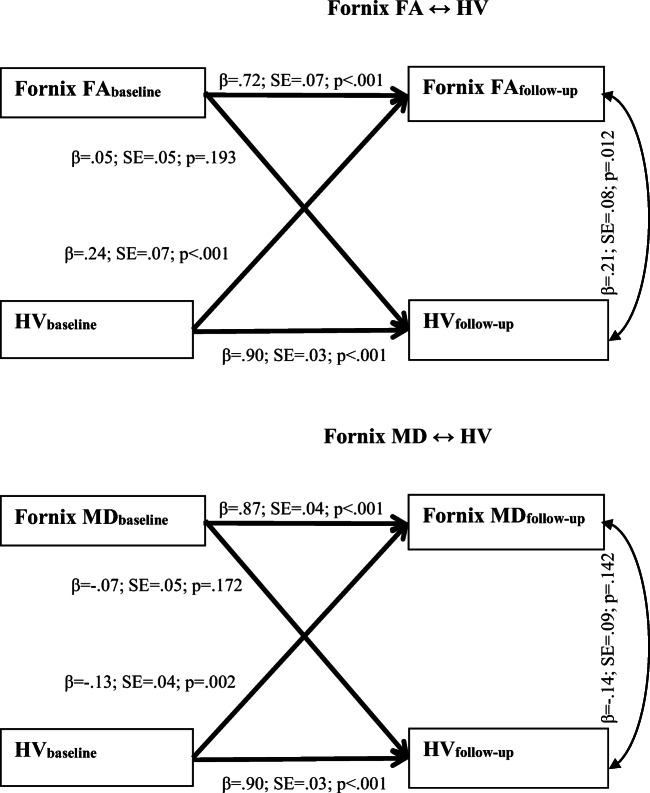
Robust Maximum likelihood estimation of the cross-lagged effects between extracted regions of interest and hippocampal volume, with age, sex and education included as covariates. Straight lines represent regression paths. Curve line represents residual covariance. FA = Fractional Anisotropy; MD = Mean Diffusivity; HV = Hippocampal volume (adjusted for intracranial volume); β = standardized coefficients; SE = Standard Error

Figure 3 presents the results of the cross-lagged analyses between HV and fornix WM indices. Just as before, all autoregressive paths were highly significant (*β*s > .72; *p*s < .001). HVs at baseline significantly predicted fornix FA and MD in their expected directions (*p*s ≤ .002), after controlling for baseline fornix FA and MD respectively. The reverse associations were not significant. Fornix FA and MD were not significantly associated with follow-up HVs (*p*s ≥ .172), after controlling for baseline HVs.

### Sub-analyses

To test the robustness of our findings, additional sets of cross-lagged analyses were carried out within various subgroups. For these analyses, we report only the directional associations that have altered in statistical significance relative to the original analyses (i.e., from significant to non-significant or vice-versa) for brevity. The detailed results of these analyses are presented in the supplementary materials (see table s1 and fig s5–13).

The first set of analyses excluded the participants who were cognitively normal, non-converters, Aβ- and APOε4+ (N_analyzed_ = 108). The results revealed that the ADNI-MEM → Fornix MD association had dropped out in statistical significance (from *p* = .046 to *p* = .148) (see fig). Furthermore, the fit indices for the Fornix FA ↔ HV model had missed the acceptable threshold marginally, resulting in a marginally acceptable fit (see Table S1).

The second set excluded participants who were both Aβ- and APOε4-, regardless of their neurocognitive or conversion status (N_analyzed_ = 94). Similarly, the ADNI-MEM → Fornix MD association had dropped out in statistical significance, though very marginally (from p = .046 to *p* = .058) (see fig. S7).

To further examine the study’s hypotheses among individuals with significant evidence of AD pathophysiological process, we identified 68 participants among all Aβ + participants, who were CSF tau positive (p-tau ≥23 pg/ml (Shaw et al. 2009)) and entered them into the same analyses. In these results, the previously non-significant Fornix FA → HV association had become statistically significant (from *p* = .193 to *p* = .025) (see fig. S10).

Relatedly, we also carried out the analyses among participants have little or no evidence of the AD pathophysiological process. These are participants who were Aβ- regardless of their neurocognitive, conversion or APOε4 status (N_analyzed_ = 40). There were major differences between the results of these analyses and those of the original. The ADNI-MEM → Fornix MD, ADNI-MEM → Fornix FA and HV → Fornix MD associations were no longer statistically significant (ps ≥ .091), whereas the previously non-significant association of Fornix MD → HV had become statistically significant (*p* = .172 to *p* = .027) (see fig. S11 and S12).

Finally, we controlled for participants’ baseline neurocognitive status (i.e., cognitive normal or MCI due to AD) in cross-lagged analyses of Fornix FA↔HV and Fornix MD↔HV, by including them as covariates. No participants were excluded in this set of analyses. (see fig. S13). Relative to the original analyses, the statistical significance for all directional associations were similar (see fig. S13). We did not repeat the analyses of Fornix FA↔ADNI-MEM and Fornix MD↔ADNI-MEM to control for neurocognitive status since such diagnoses were largely contingent on the memory scores.

## Discussion

We have demonstrated the directional relationships between the microstructure of the fornix, verbal memory, and hippocampal volume in the context of AD progression. These findings do not support our bidirectional hypothesis of disrupted fornix microstructure and memory impairments. While impaired verbal memory and smaller hippocampal volumes at baseline significantly predicted worse fornix microstructure two years later, the reverse direction of associations were not found to be significant. Baseline fornix microstructure was not significantly associated with subsequent memory impairment or hippocampal volumes. Our findings did however support the second hypothesis on hippocampal atrophy predicting the microstructure of the fornix at follow-up. It should be noted that these finding are unlikely to be driven by baseline differences in memory, fornix microstructure or HV, as result of diagnosis status or other factors, since these baseline variables are already controlled for in our autoregressive models.

We carried out additional analyses to examine if such associations hold within various subgroups. The results of these analyses were largely similar to our main findings, with some interesting exceptions. First, among the group of individuals with significant AD pathophysiology, there was a significant bidirectional relationship between fornix FA and HV. Fornix FA predicted subsequent HV and vice versa. These findings suggest that the axial or radial degeneration of fornix WM tracts (but not both, hence impacting FA more than MD), tends to be tightly coupled with hippocampal atrophy. Next, among participants with little or no AD pathophysiology, there were no significant associations between fornix FA/MD and memory in any direction. Relative to the main analyses, the directional association between fornix MD and HV had reversed, while that of fornix FA and HV had remained the same. These findings suggest the combined axial and radial degeneration (i.e., MD) of fornix WM fibers preceded hippocampal atrophy in the early stages. Then, following significant hippocampal atrophy, the fornix WM fiber tracts continued to degenerate, however, only in an axial or radial manner to influence FA more so than MD. Nevertheless, regardless of the type of fornix WM degeneration, the changes fornix microstructure did not appear to be related to memory decline. The findings of these two sets of analyses taken together would very roughly suggest that AD pathophysiology accelerates hippocampal atrophy and memory decline more so than the deterioration of fornix microstructure, especially in that of MD. An alternative or perhaps less plausible interpretation would be that AD pathophysiology delays alterations in fornix microstructure, until significant memory decline and hippocampal atrophy have occurred. Given the small sample sizes associated with these analyses, these interpretations are rudimentary at best and should be clarified with larger sampled studies.

Our findings from the main analyses are consistent with Fletcher et al.'s (2013) observations that fornix FA do not significantly predict subsequent conversion to aMCI or AD). In another similar study, Zhuang et al.'s (2012) whole brain tract-based spatial statistics revealed that aMCI converters had significantly lower fornix FA at baseline. Nevertheless, it was not predictive of subsequent verbal episodic memory in their multiple regression models. Such conflicting findings, within a study, alluded to the idea that fornix FA may not be a robust predictor of subsequent memory decline in general. Likewise, although Mielke et al. (2012) documented a significant association between baseline fornix FA and subsequent memory impairment, similar to the present study, the magnitude of such association was reportedly very small (standardized coefficient = .01) compared to that of HV and subsequent memory impairment. Our results instead provide support for the reverse association – memory impairments significantly predict subsequent disruption in fornix microstructure. This is consistent with Nowrangi et al.'s (2013) report of individuals with aMCI or AD, relative to healthy controls, presenting with significantly increased fornix MD over time. Although Teipel et al. (2014) did not report a significant fornix FA decline in their follow-up of participants with aMCI relative to healthy controls, the absolute decline in FA in the former group was approximately four times larger than the latter. Given the small sample size, it is possible that their study was underpowered to detect a significant time by group interaction. Overall, the accumulated evidence suggests memory impairment precedes changes in fornix microstructure.

In relation to the second hypothesis, Fletcher et al. (2013) previously reported cross-sectional associations between HV and fornix microstructure. Our novel longitudinal findings go one step further to illustrate the directional nature of such associations. We found that smaller HVs were associated with worse fornix microstructure at follow-up, however, the reverse association (i.e. fornix microstructure predicting subsequent HVs) was not significant. Relatedly, a previous longitudinal study illustrated a similar sequential relationship between hippocampal atrophy and WM pathology with baseline hippocampal volumes predicting subsequent WM atrophy in the cingulum and uncinate fasciculus (Villain et al. 2010). These findings converge to suggest WM pathology is likely to appear in the late prodromal stage of AD where there has already been a noticeable decline in memory and HV.

### Insights into the mechanisms of AD progression in humans

Our findings advance the current understanding of the pathophysiological processes involved in the early prodromal stages of AD. The observation of hippocampal atrophy predicting subsequent disruption in fornix microstructure is consistent with Kantarci's (2014) proposal of hippocampal neuronal death preceding subsequent degeneration of fornix WM fiber tracts. Along with the fact that hippocampal atrophy predicted subsequent memory impairment, these findings provide support for the amyloid cascade hypothesis. The possible notion that AD pathophysiology accelerates hippocampal atrophy and memory decline more so than the deterioration of fornix microstructure, as alluded to above, would also be consistent with such a hypothesis. On the other hand, given that fornix microstructure did not significantly predict subsequent memory impairment, such memory impairment is unlikely to be associated with a WM-related pathology, at least in the fornix. Taken together, these findings cast doubt on the possibility of neuroinflammatory processes implicating the fornix in the pathogenesis of AD. Nevertheless, we cannot rule out the possibility of neuroinflammatory processes acting on other WM regions commonly observed to be altered in prodromal stages of AD apart from the fornix, such as the uncinate fasciculus and parahippocampal cingulum (Yu et al. 2017). Future longitudinal research may consider employing a whole-brain approach to investigate if certain memory-associated WM microstructural alterations in the brain are independent, or even precede, hippocampal atrophy to examine such a possibility.

### Significant clinical implications

Results of our study provide important information in regard to the use of neural markers in the context of prodromal AD. Previous cross-sectional studies have frequently observed altered fornix microstructure in the prodromal stages of AD, and on the basis of this cross-sectional evidence some have concluded that this pathology represents an early marker of AD (for a review see Nowrangi and Rosenberg (2015)). Whilst this conclusion is not wrong, our findings suggest it is not entirely correct. Our observation of fornix microstructure being predicted by preceding hippocampal atrophy and memory impairment suggests that fornix microstructural alterations do not occur early enough in the course of prodromal AD; these alterations may indicate an individual to be in a late prodromal stage of AD. As such, if fornix microstructure was used as an early marker in the assessment of AD, one would have incorrectly identified individuals in the late prodromal stage as belonging to the early stage. The implication of this inaccurate identification cannot be understated especially given that the best course of treatment in AD largely depends on the stage of the disease (Anand et al. 2014). In tracking the early progression of AD, hippocampal volumetric measurements, relative to fornix microstructural indices, are perhaps the more useful non-invasive neural markers given their value in predicting future memory decline. Next, the current study together with a previous work (Villain et al. 2010) demonstrates the sequential nature of GM and WM pathology. Based on this information, one could chart the progression of AD more confidently via the use of multiple GM and WM markers to better inform the optimal course of treatment.

### Limitations

In the current study, we did not carry out free water elimination (Pasternak et al. 2009), as such the fornix WM indices may have been susceptible to CSF contamination, especially given its intraventricular location. Nevertheless, we have attempted to minimize such partial volume effects by only including voxels in the thresholded mean FA skeleton into the fornix ROI. Next, our interpretation of the study’s findings on AD related pathophysiological process is limited by the fact that not all of these participants would eventually develop AD in their lifetime. Strengths of the current study include a relatively large sample size and consistency in the effects relating to both DTI indices for the main analyses. The latter alluded to the robustness of our results given that previous studies in this area have generally found significant effects in one DTI measure, but not the other.

## Conclusions

The current study sought to evaluate the utility of fornix microstructure as an early neural marker of AD by examining its longitudinal relationship with memory impairment and hippocampal atrophy. We found that memory impairment and HVs significantly predicted subsequent disruption in fornix microstructure. However, fornix microstructure was not significantly associated with subsequent memory impairment and HVs. These results suggest disruptions in fornix microstructure are likely to occur in the late prodromal AD stage, where significant memory impairment and hippocampal atrophy has already occurred. These longitudinal findings are crucial in refining the use of these neural markers in the early detection of AD and advancing our understanding of development of AD.

## Electronic supplementary material

ESM 1 (DOCX 926 kb)
