# Supplementary material for: Verbal memory and hippocampal volume predict subsequent fornix microstructure in those at risk for Alzheimer’s disease
Source: Brain Imaging Behav. 2019 Sep 7;14(6):2311–22. doi: 10.1007/s11682-019-00183-8 (PMC7647989; doi:10.1007/s11682-019-00183-8)
Supplement: Supplementary file 1 — (DOCX 926 kb) [file 11682_2019_183_MOESM1_ESM.docx]

| 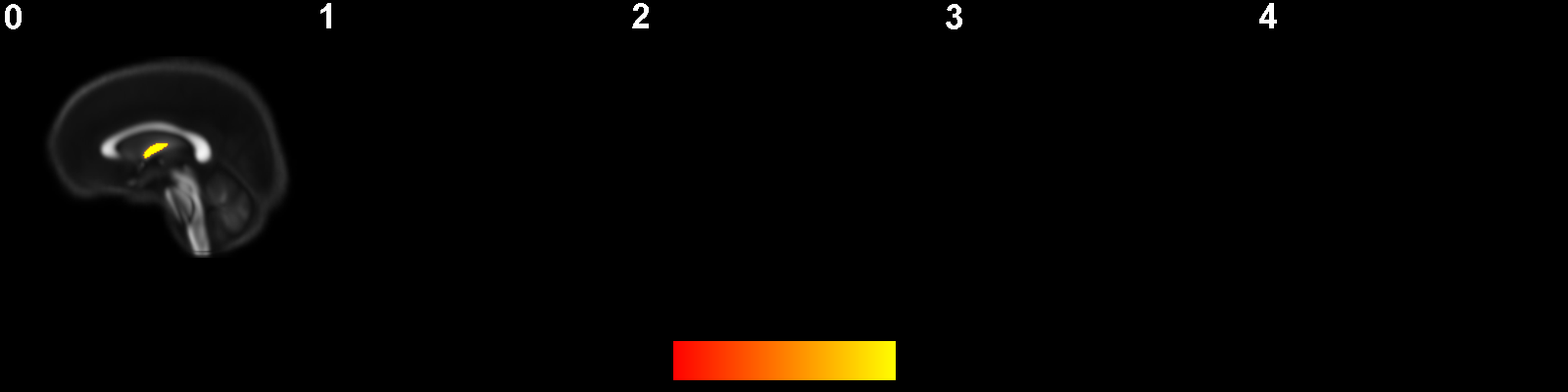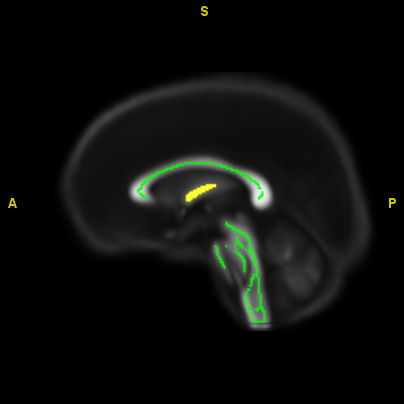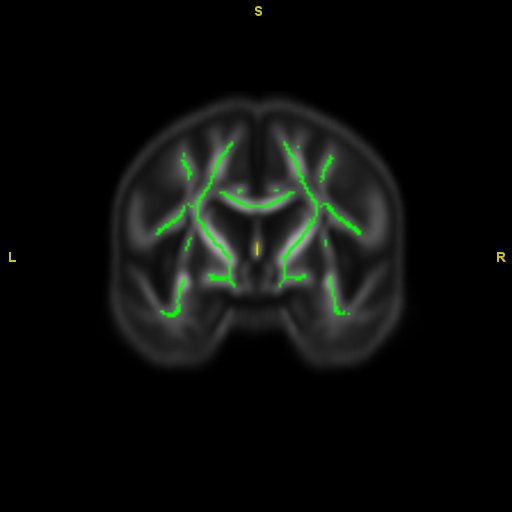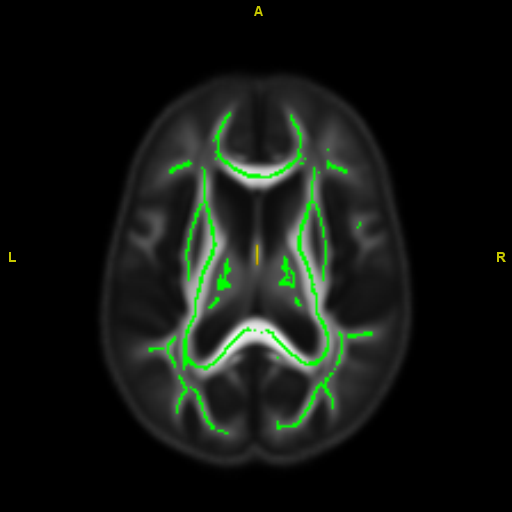  **Z= 25.5**  **100**  **33**  **%overlap**  **X= -78**  **Y= 37.5** |
| --- |

**Fig S1.** Fornix ROI overlaid on the mean FA image. The color bar range indicates the degree of non-zero voxels overlap across participants.

| 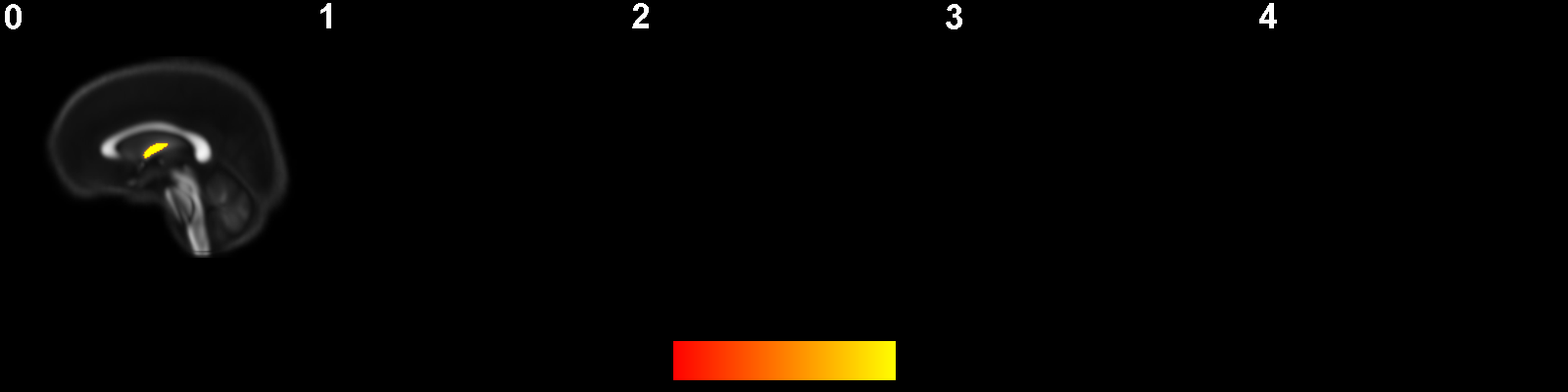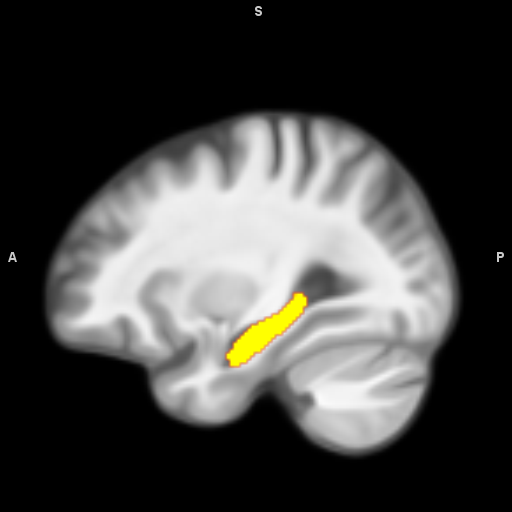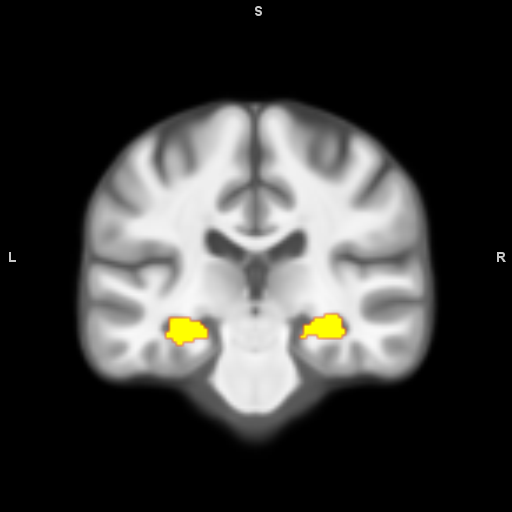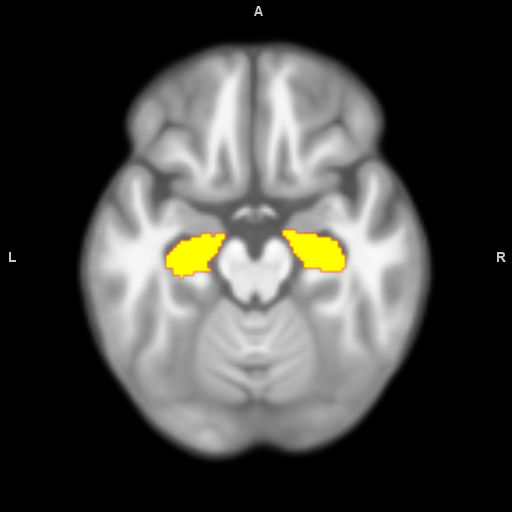  **%overlap**  **33**  **Y= -25.5**  **X= 30**  **Z= -18**  **100**  **%overlap** |
| --- |

**Fig S2.** Hippocampus ROI overlaid on the mean brain image. The color bar range indicates the degree of non-zero voxels overlap across participants.

| 1. **ADNI-MEM scores** | |
| --- | --- |
| **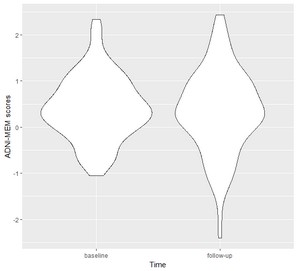** | **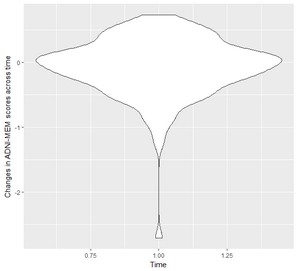** |
| 1. **Fornix FA** | |
| **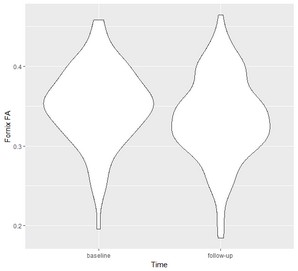** | **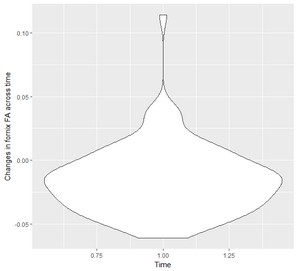** |
|  |  |
| 1. **Fornix MD** | |
| **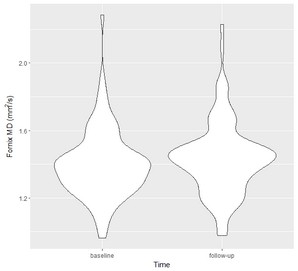** | **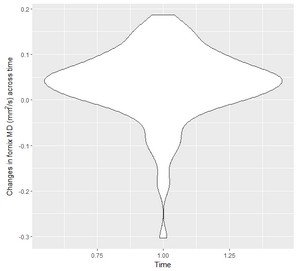** |
| 1. **Hippocampal volume** | |
| **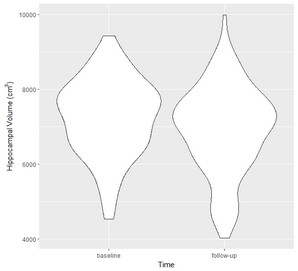** | **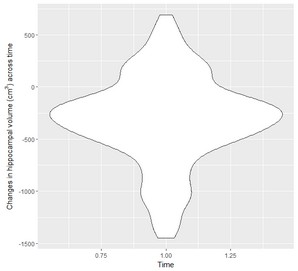** |
|  | |
|  |  |

**Fig S3**. Violin plots showing baseline, follow up and difference across time (follow-up – baseline) of a) ADNI-MEM scores b) fornix fractional anisotropy, c) fornix mean diffusivity, and d) hippocampal volume.

**HV ↔ ADNI-MEM**

**ADNI-MEM_baseline_**

**HV_baseline_**

**HV_follow-up_**

**ADNI-MEM_follow-up_**

β=.89; SE=.03; p<.001

β=.86; SE=.04; p<.001

β=.10; SE=.04; p=.008

β=.14; SE=.05; p=.007

β=-.04; SE=.08; p=.622

**Fig S4**. Robust Maximum likelihood estimation of the cross-lagged effects between extracted regions of interest and ADNI-MEM, with age, sex and education included as covariates. Straight lines represent regression paths. Curve lines represent residual covariance. FA = Fractional Anisotropy; MD = Mean Diffusivity; HV = Hippocampal volume (adjusted for intracranial volume); β = standardized coefficients; SE = Standard Error. Model fit: CFI= .963; SRMR= .093.

**Sub-analyses**

**Table S1.** Fit indices of all examined SEM models

| Model | Model | CFI | SRMR |
| --- | --- | --- | --- |
| Excluding participants who are CN, NC, Aβ- and APOε4+ (N=108) |  |  |  |
|  | Fornix FA ↔ ADNI-MEM | .993 | .034 |
|  | Fornix MD ↔ ADNI-MEM | .996 | .033 |
|  | Fornix FA ↔ HV | .947 | .105 |
|  | Fornix MD ↔ HV | .960 | .093 |
| Excluding participants who are Aβ- and APOε4-, regardless of conversion or neurocognitive status (N=94) |  |  |  |
|  | Fornix FA ↔ ADNI-MEM | .994 | .036 |
|  | Fornix MD ↔ ADNI-MEM | .994 | .039 |
|  | Fornix FA ↔ HV | .967 | .077 |
|  | Fornix MD ↔ HV | .965 | .088 |
| All Aβ+ participants (N=68) |  |  |  |
|  | Fornix FA ↔ ADNI-MEM | .998 | .032 |
|  | Fornix MD ↔ ADNI-MEM | .995 | .032 |
|  | Fornix FA ↔ HV | .966 | .088 |
|  | Fornix MD ↔ HV | .970 | .091 |
| All Aβ- participants (N=40) |  |  |  |
|  | Fornix FA ↔ ADNI-MEM | .989 | .039 |
|  | Fornix MD ↔ ADNI-MEM | .999 | .029 |
|  | Fornix FA ↔ HV | .976 | .070 |
|  | Fornix MD ↔ HV | .942 | .092 |
| Including baseline neurocognitive status (MCI due to AD or CN) as covariates (N=115) |  |  |  |
|  | Fornix FA ↔ HV | .950 | .095 |
|  | Fornix MD ↔ HV |  |  |

*Note.* CN= cognitively normal; NC= non-converters; Aβ*-* = CSF amyloid-beta negative; APOε4+= APOε4 positive; APOε4-= APOε4 negative; CFI = Comparative Fit Index; SRMR=Standardized Root Mean Square residual; FA= Fractional Anisotropy; MD = Mean Diffusivity; HV= Hippocampal volume (adjusted for intracranial volume); ADNI-MEM= memory composite scores.

**Excluding participants who are CN, NC, Aβ- and APOε4+:**

**Fornix FA ↔ ADNI-MEM**

**ADNI-MEM_baseline_**

**Fornix FA_baseline_**

**Fornix FA_follow-up_**

**ADNI-MEM_follow-up_**

β=.82; SE=.04; p<.001

β=.86; SE=.06; p<.001

β=.10; SE=.05; p=.024

β=--.01; SE=.05; p=.813

β=.13; SE=.09; p=.140

**Fornix MD ↔ ADNI-MEM**

**ADNI-MEM_baseline_**

**Fornix MD_baseline_**

**Fornix MD_follow-up_**

**ADNI-MEM_follow-up_**

β=.91; SE=.03; p<.001

β=.86; SE=.04; p<.001

β=-.05; SE=.04; p=.148

β=-.02; SE=.05; p=.751

β=-.07; SE=.10; p=.499

**Fig S5.** Robust Maximum likelihood estimation of the cross-lagged effects between extracted regions of interest and ADNI-MEM, with age, sex and education included as covariates. Straight lines represent regression paths. Curve lines represent residual covariance. FA = Fractional Anisotropy; MD = Mean Diffusivity; β = standardized coefficients; SE = Standard Error.

**Fornix FA ↔ HV**

**Fornix FA_baseline_**

**HV_baseline_**

**HV_follow-up_**

**Fornix FA_follow-up_**

β=.90; SE=.03; p<.001

β=.79; SE=.05; p<.001

β=.17; SE=.06; p=.006

β=.05; SE=.04; p=.305

β=.25; SE=.10; p=.009

**Fornix MD ↔ HV**

**Fornix MD_baseline_**

**HV_baseline_**

**HV_follow-up_**

**Fornix MD_follow-up_**

β=.90; SE=.03; p<.001

β=.90; SE=.03; p<.001

β=-.09; SE=.04; p=.012

β=-.07; SE=.05; p=.222

β=-.15; SE=.11; p=.151

**Fig S6.** Robust Maximum likelihood estimation of the cross-lagged effects between extracted regions of interest and ADNI-MEM, with age, sex and education included as covariates. Straight lines represent regression paths. Curve line represents residual covariance. FA = Fractional Anisotropy; MD = Mean Diffusivity; HV = Hippocampal volume (adjusted for intracranial volume); β = standardized coefficients; SE = Standard Error.

**Excluding participants who are Aβ- and APOε4-, regardless of conversion or neurocognitive status**

**Fornix FA ↔ ADNI-MEM**

**ADNI-MEM_baseline_**

**Fornix FA_baseline_**

**Fornix FA_follow-up_**

**ADNI-MEM_follow-up_**

β=.76; SE=.06; p<.001

β=.87; SE=.04; p<.001

β=.12; SE=.06; p=.027

β=--.02; SE=.05; p=.751

β=.07; SE=.09; p=.418

**Fornix MD ↔ ADNI-MEM**

**ADNI-MEM_baseline_**

**Fornix MD_baseline_**

**Fornix MD_follow-up_**

**ADNI-MEM_follow-up_**

β=.90; SE=.03; p<.001

β=.91; SE=.03; p<.001

β=-.07; SE=.04; p=.058

β=-.04; SE=.06; p=.433

β=-.12; SE=.16; p=.456

**Fig S7.** Robust Maximum likelihood estimation of the cross-lagged effects between extracted regions of interest and ADNI-MEM, with age, sex and education included as covariates. Straight lines represent regression paths. Curve lines represent residual covariance. FA = Fractional Anisotropy; MD = Mean Diffusivity; β = standardized coefficients; SE = Standard Error.

**Fornix FA ↔ HV**

**Fornix FA_baseline_**

**HV_baseline_**

**HV_follow-up_**

**Fornix FA_follow-up_**

β=.90; SE=.03; p<.001

β=.73; SE=.07; p<.001

β=.24; SE=.07; p=.001

β=.09; SE=.05; p=.064

β=.09; SE=.10; p=.328

**Fornix MD ↔ HV**

**Fornix MD_baseline_**

**HV_baseline_**

**HV_follow-up_**

**Fornix MD_follow-up_**

β=.90; SE=.03; p<.001

β=.87; SE=.04; p<.001

β=-.15; SE=.05; p=.004

β=-.08; SE=.06; p=.219

β=-.07; SE=.10; p=.510

**Fig S8.** Robust Maximum likelihood estimation of the cross-lagged effects between extracted regions of interest and ADNI-MEM, with age, sex and education included as covariates. Straight lines represent regression paths. Curve line represents residual covariance. FA = Fractional Anisotropy; MD = Mean Diffusivity; HV = Hippocampal volume (adjusted for intracranial volume); β = standardized coefficients; SE = Standard Error.

**Aβ+ and p-tau+ participants:**

**Fornix FA ↔ ADNI-MEM**

**ADNI-MEM_baseline_**

**Fornix FA_baseline_**

**Fornix FA_follow-up_**

**ADNI-MEM_follow-up_**

β=.75; SE=.05; p<.001

β=.90; SE=.03; p<.001

β=.13; SE=.05; p=.005

β=-01; SE=.07; p=.925

β=.31; SE=.13; p=.015

**Fornix MD ↔ ADNI-MEM**

**ADNI-MEM_baseline_**

**Fornix MD_baseline_**

**Fornix MD_follow-up_**

**ADNI-MEM_follow-up_**

β=.87; SE=.05; p<.001

β=.90; SE=.03; p<.001

β=-.10; SE=.05; p=.049

β=-.10; SE=.08; p=.209

β=-.17; SE=.17; p=.324

**Fig S9.** Robust Maximum likelihood estimation of the cross-lagged effects between extracted regions of interest and ADNI-MEM, with age, sex and education included as covariates. Straight lines represent regression paths. Curve lines represent residual covariance. FA = Fractional Anisotropy; MD = Mean Diffusivity; β = standardized coefficients; SE = Standard Error.

**Fornix FA ↔ HV**

**Fornix FA_baseline_**

**HV_baseline_**

**HV_follow-up_**

**Fornix FA_follow-up_**

β=.90; SE=.04; p<.001

β=.74; SE=.06; p<.001

β=.17; SE=.09; p=.049

β=.15; SE=.07; p=.025

β=.28; SE=.12; p=.015

**Fornix MD ↔ HV**

**Fornix MD_baseline_**

**HV_baseline_**

**HV_follow-up_**

**Fornix MD_follow-up_**

β=.89; SE=.04; p<.001

β=.85; SE=.05; p<.001

β=-.12; SE=.06; p=.041

β=-.16; SE=.09; p=.084

β=-.22; SE=.12; p=.053

**Fig S10.** Robust Maximum likelihood estimation of the cross-lagged effects between extracted regions of interest and ADNI-MEM, with age, sex and education included as covariates. Straight lines represent regression paths. Curve line represents residual covariance. FA = Fractional Anisotropy; MD = Mean Diffusivity; HV = Hippocampal volume (adjusted for intracranial volume); β = standardized coefficients; SE = Standard Error.

**All Aβ- participants:**

**Fornix FA ↔ ADNI-MEM**

**ADNI-MEM_baseline_**

**Fornix FA_baseline_**

**Fornix FA_follow-up_**

**ADNI-MEM_follow-up_**

β=.669; SE=.17; p<.001

β=.77; SE=.11; p<.001

β=.11; SE=.12; p=.347

β=.01; SE=.10; p=.944

β=-.03; SE=.10; p=.782

**Fornix MD ↔ ADNI-MEM**

**ADNI-MEM_baseline_**

**Fornix MD_baseline_**

**Fornix MD_follow-up_**

**ADNI-MEM_follow-up_**

β=.87; SE=.11; p<.001

β=.87; SE=.04; p<.001

β=-.07; SE=.09; p=.427

β=.06; SE=.11; p=.567

β=-.07; SE=.10; p=.499

**Fig S11.** Robust Maximum likelihood estimation of the cross-lagged effects between extracted regions of interest and ADNI-MEM, with age, sex and education included as covariates. Straight lines represent regression paths. Curve lines represent residual covariance. FA = Fractional Anisotropy; MD = Mean Diffusivity; β = standardized coefficients; SE = Standard Error.

**Fornix FA ↔ HV**

**Fornix FA_baseline_**

**HV_baseline_**

**HV_follow-up_**

**Fornix FA_follow-up_**

β=.92; SE=.05; p<.001

β=.52; SE=.20; p=.010

β=.40; SE=.12; p=.001

β=.08; SE=.06; p=.214

β=.32; SE=.15; p=.030

**Fornix MD ↔ HV**

**Fornix MD_baseline_**

**HV_baseline_**

**HV_follow-up_**

**Fornix MD_follow-up_**

β=.91; SE=.05; p<.001

β=.82; SE=.14; p<.001

β=-.16; SE=.09; p=.091

β=-.17; SE=.078; p=.027

β=-.13; SE=.16; p=.426

**Fig S12.** Robust Maximum likelihood estimation of the cross-lagged effects between extracted regions of interest and ADNI-MEM, with age, sex and education included as covariates. Straight lines represent regression paths. Curve line represents residual covariance. FA = Fractional Anisotropy; MD = Mean Diffusivity; HV = Hippocampal volume (adjusted for intracranial volume); β = standardized coefficients; SE = Standard Error.

**Including baseline neurocognitive status (MCI due to AD or CN) as covariates:**

**Fornix FA ↔ HV**

**Fornix FA_baseline_**

**HV_baseline_**

**HV_follow-up_**

**Fornix FA_follow-up_**

β=.90; SE=.03; p<.001

β=.72; SE=.07; p<.001

β=.24; SE=.07; p<.001

β=.05; SE=.04; p=.193

β=.21; SE=.08; p=.012

**Fornix MD ↔ HV**

**Fornix MD_baseline_**

**HV_baseline_**

**HV_follow-up_**

**Fornix MD_follow-up_**

β=.90; SE=.03; p<.001

β=.87; SE=.04; p<.001

β=-.13; SE=.04; p=.002

β=-.07; SE=.05; p=.172

β=-.13; SE=.16; p=.426

**Fig S13.** Robust Maximum likelihood estimation of the cross-lagged effects between extracted regions of interest and ADNI-MEM, with age, sex and education included as covariates. Straight lines represent regression paths. Curve line represents residual covariance. FA = Fractional Anisotropy; MD = Mean Diffusivity; HV = Hippocampal volume (adjusted for intracranial volume); β = standardized coefficients; SE = Standard Error.
